# Frontal midline theta differentiates separate cognitive control strategies while still generalizing the need for cognitive control

**DOI:** 10.1038/s41598-021-94162-z

**Published:** 2021-07-19

**Authors:** Jarrod Eisma, Eric Rawls, Stephanie Long, Russell Mach, Connie Lamm

**Affiliations:** 1 Department of Radiology and Radiological Sciences, Vanderbilt University Medical Center, Nashville, USA; 2 Department of Psychiatry and Behavioral Sciences, University of Minnesota Health, Minneapolis, MN 55414 USA; 3 Department of Psychological Sciences, University of Arkansas, Fayetteville, USA

**Keywords:** Cognitive control, Human behaviour

## Abstract

Cognitive control processes encompass many distinct components, including response inhibition (stopping a prepotent response), proactive control (using prior information to enact control), reactive control (last-minute changing of a prepotent response), and conflict monitoring (choosing between two competing responses). While frontal midline theta activity is theorized to be a general marker of the need for cognitive control, a stringent test of this hypothesis would require a quantitative, within-subject comparison of the neural activation patterns indexing many different cognitive control strategies, an experiment lacking in the current literature. We recorded EEG from 176 participants as they performed tasks that tested inhibitory control (Go/Nogo Task), proactive and reactive control (AX-Continuous Performance Task), and resolving response conflict (Global/Local Task-modified Flanker Task). As activity in the theta (4–8 Hz) frequency band is thought to be a common signature of cognitive control, we assessed frontal midline theta activation underlying each cognitive control strategy. In all strategies, we found higher frontal midline theta power for trials that required more cognitive control (target conditions) versus control conditions. Additionally, reactive control and inhibitory control had higher theta power than proactive control and response conflict, and proactive control had higher theta power than response conflict. Using decoding analyses, we were able to successfully decode control from target trials using classifiers trained exclusively on each of the other strategies, thus firmly demonstrating that theta representations of cognitive control generalize across multiple cognitive control strategies. Our results confirm that frontal midline theta-band activity is a common mechanism for initiating and executing cognitive control, but theta power also differentiates between cognitive control mechanisms. As theta activation reliably differs depending on the cognitive control strategy employed, future work will need to focus on the differential role of theta in differing cognitive control strategies.

## Introduction

Cognitive control processes are activated in the brain when habitual neuronal responses are inadequate to support goal-oriented behavior^1,2^. Cognitive control is an umbrella term that encompasses many distinct subcomponents^3^. These subcomponents of cognitive control are typically assessed using various laboratory paradigms that require withholding a predominant response (response inhibition^4^), paradigms that induce conflict at the stimulus level by priming multiple competing responses (response conflict^5–7^), or continuous performance tasks that differentiate just-in-time (reactive) control and planned (proactive) control^8,9^. While cognitive control processes are thought to rely on a core set of brain mechanisms^10^, a comparison of brain activity giving rise to each of these fundamentally different cognitive control strategies has yet to be completed. Several studies have compared neural activation during different tasks (Go/Nogo vs. Stop-Signal^11^; AX-Continuous Performance Task versus Dot Pattern Expectancy^12^), but these comparisons were limited to tasks enacting the same control strategy (inhibitory control and proactive/reactive control, respectively). Several other studies^13,14^ have examined neural signatures of cognitive control across multiple strategies. Cavanagh and colleagues^13^ examined patterns of theta activation using tasks tapping response inhibition (Oddball) and response conflict (Cued Simon Task) strategies, and Nigbur and colleagues^14^ examined patterns of theta activation using a response inhibition task (Go/Nogo) and two different response conflict-inducing tasks (Flanker and Simon tasks). However, neither of these included the AX-Continuous performance task, or an analogue thereof. Thus, previous comparisons of theta activation patterns across multiple cognitive control strategies did not assess participants’ proactive and reactive control.

To better understand cognitive control processes, researchers investigate their underlying neural mechanisms. In particular, electroencephalography (EEG) is used for its temporal resolution to quantify and characterize the dendritic potentials of the brain’s upper cortex. Electrophysiological oscillations play a role in eliciting cognitive control^15^, and EEG can record and quantify these oscillations due to its high temporal precision. Cognitive control is implemented by neocortical structures^16,17^, and EEG is sensitive to time-resolved computations in the neocortex. One frequent form of EEG analysis uses event-related potentials (ERPs) in the time domain, comprising the average phase-locked voltage deflections recorded across experimental trials. In particular, the N2 (the negative deflection peaking between 200 and 400 ms post-stimulus in frontal midline scalp regions) increases in magnitude during high conflict trials compared to low-conflict trials in go/nogo tasks^11,18^, Eriksen flanker tasks^19,20^, AX-continuous performance tasks (AX-CPT)^21–23^, and stop-signal paradigms^11,24^, all situations requiring cognitive control^23,25^. However, ERPs collapse the time-domain signal across the frequency bands, thereby removing the frequency variations of the signal, and ERPs examine only the phase-locked signal captured by the EEG.

Time–frequency analysis profiles the changes in spectral power (both phase-locked and non-phase-locked) of the EEG recording, finely characterizing frequency-specific task-relevant neural computations. Time–frequency analysis decomposes brain signals into a complex amplitude/phase space, and therefore can also measure the consistency of oscillatory phase angles across trials. By providing information about the timing of frequency-specific changes in power and phase consistency with respect to stimulus or response onset, time–frequency analysis poses an advantage to ERP analysis. The detailed information provided by time–frequency analysis is powerful in studying cognitive control, because oscillatory activity of neuron populations appears to be a fundamental mechanism that coordinates brain networks during the need for cognitive control^1,2,21,26,27^. The need for cognitive control preferentially modulates EEG activity in the theta (4–8 Hz) frequency band^2,13,15,28^. One source of increased theta power during cognitive control is believed to be the anterior cingulate cortex (ACC)^1,13,29,30^. Frontal midline theta has also been more broadly implicated in the medial prefrontal cortex (mPFC), using combined EEG and magnetoencephalography (MEG) source-modeled data^31,32^. Previous comparison of multiple cognitive control strategies has also suggested that frontal theta might have different generators in different cognitive control strategies^14^. However, previous source localized analyses generally preclude the influence of more spatially distant brain regions (i.e. other than medial/lateral frontal cortex).

The current study aimed to confirm the perspective that frontal midline theta is a general substrate underlying cognitive control^13^, and to provide a comparative analysis on the theta profiles of four distinct cognitive control strategies: proactive control, reactive control, inhibitory control, and conflict monitoring. The inclusion of all four cognitive control strategies within the same sample was an entirely novel feature of this study. No published research to date has measured proactive and reactive control simultaneously with the two other strategies (i.e., inhibitory control, conflict monitoring). In particular, the inclusion of a cognitive control strategy that explicitly required planning of future control (proactive control) is important, as previous comparisons across multiple cognitive control strategies have all required just-in-time cognitive control. Inhibitory control was operationalized using a Go/Nogo task^4^. ACC plays a role in successful response inhibition, through making and monitoring decisions^13,30,33^. Several reviews propose that frontal midline theta activation is an underlying mechanism of response inhibition^1,2,13^. Proactive and reactive control were operationalized through the AX-Continuous Performance Task^9^. Proactive control processes prepare the brain to be particularly sensitive to incoming goal-relevant stimuli, and reactive control processes are more reactionary mechanisms that resolve conflict and overcome interference^21^. Both reactive and proactive control mechanisms depend on theta frontoparietal oscillatory networks^21^, supporting the notion that frontal midline theta oscillations are a key mechanism to enacting cognitive control. Response conflict was operationalized through a modified Eriksen Flanker task^5^ that uses the Navon Letters^34^ to elicit response conflict (letters instead of arrows). Conflict trials contained incongruent letter configurations that primed conflicting responses. Conflict monitoring and conflict resolution depend heavily on midline frontal cortex^14,15^, especially the ACC^13,29^. Numerous studies have shown that conflict monitoring is enabled by theta oscillatory networks^14,15,28^.

We hypothesized that experimental target trials, which demanded relatively more cognitive control than control trials, would elicit higher frontal midline theta power and phase-locking than control trials, in alignment with the theorized role of theta oscillations as a *lingua franca* for cognitive control^13^. While there has been a considerable amount of research delving into the bases of these separate cognitive control mechanisms, there are considerably fewer studies that were designed to contrast these cognitive control mechanisms on their time–frequency profiles within the theta range. Because of the exploratory nature of this study, we have no specific hypotheses about how various cognitive control strategies will relate to each other.

## Methods

### Participants

The EEG data for this study were collected from 176 undergraduate students in the University of Arkansas general psychology pool (Gender: 80 M, 92 F, 2 Non-binary, 2 N/A; Age: x̄ = 19.45 years, SD = 2.88 years). All participants included in this study were English-speaking and self-reported that they had no current psychiatric diagnoses or uncorrected visual impairments. Additionally, all subjects used in this study completed at least 10 correct, artifact-free trials per trial type. All students were granted course credit for their participation in this study. This study was approved by the University of Arkansas’ Institutional Review Board (IRB#: 1708026820), and all procedures were performed in accordance with the relevant guidelines and regulations. All subjects gave written informed consent prior to participating in this study.

### Procedure

For our examination of four cognitive control strategies (inhibitory control, proactive control, reactive control, and resolving response conflict), three computer-based tasks were completed by the participants. The Go/Nogo task was designed to test response inhibition. The AX continuous performance task (AX-CPT) was designed to test proactive and reactive control. The Global/Local task was designed to test one’s ability to resolve response conflict. All three tasks were presented on a 17-inch computer monitor using E-Prime software (Psychology Software Tools, Inc., Pittsburgh, Pennsylvania). Stimuli were displayed on a black screen, and each task was shuffled throughout the entire experimental trial (approximately 1.5 h). Prior to beginning the tasks themselves, participants completed two blocks of 10 practice trials each for the AX-CPT and Global/Local tasks, and one block of 10 practice trials for the Go/Nogo task.

#### Go/Nogo task (inhibitory control)

**Figure 1:**
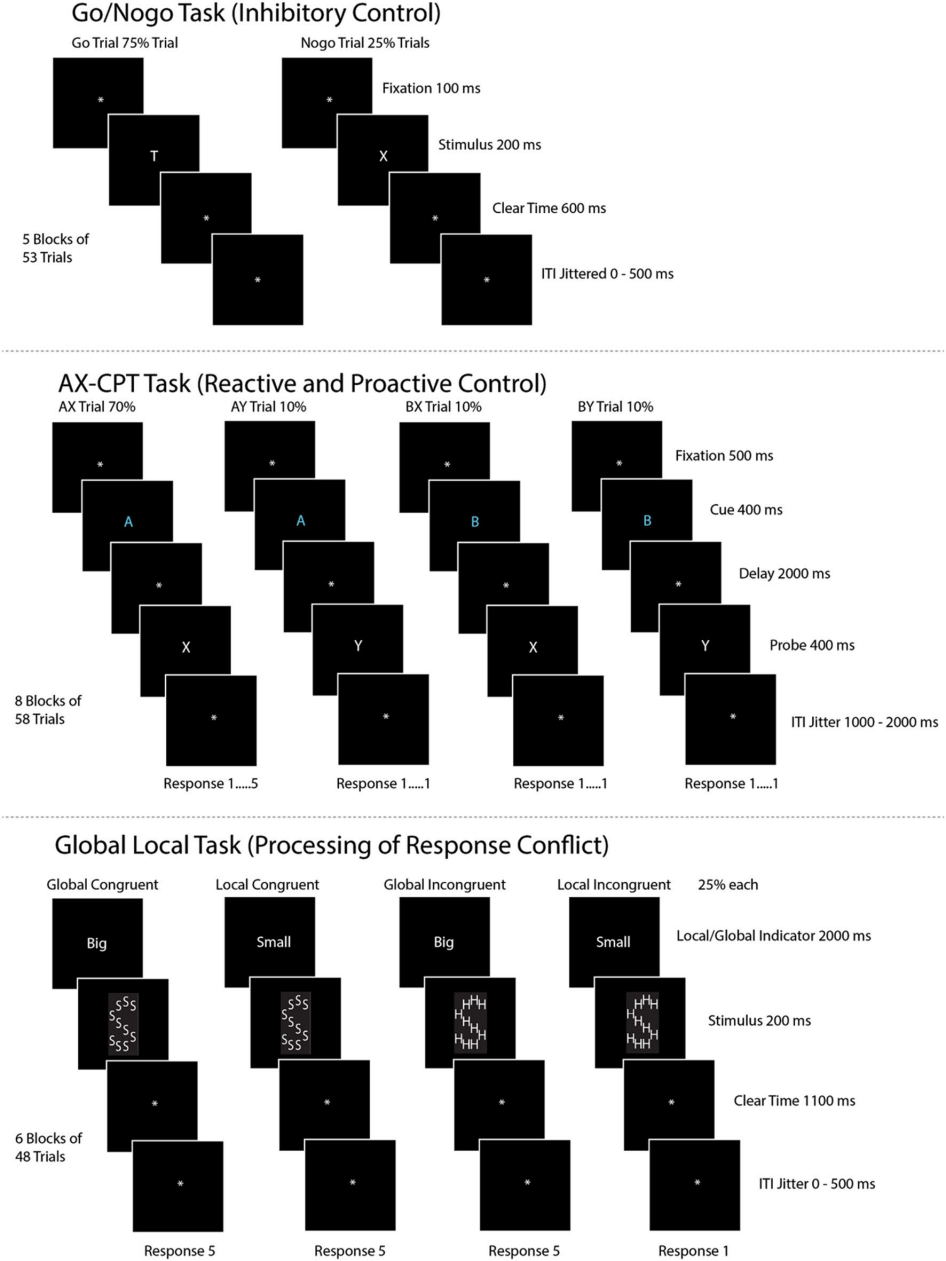
Shows stimulus order and timing information for all three cognitive control tasks.

The Go/Nogo task was adapted from the task described by Garavan and colleagues^4^. The task began with a fixation cue shown in the middle of the screen for 100 ms to focus the participant’s eyes on where the next stimulus would arrive. Stimuli consisted of a single, white letter displayed for 200 ms. Participants were instructed to respond to any letter besides the letter “X” by pressing the button labeled #1. After the stimulus cue, another fixation cue appeared for 600 ms, during which the participants responded to the stimulus cue. If the letter presented was “X”, then the participant was instructed to refrain from pressing the button (apply inhibitory control). After this fixation cue, another fixation cue was displayed for an inter-trial interval that varied from 0 to 500 ms. Go trials, in which the participant was instructed to respond, constituted 75% of the trials in this task, in order to establish a prepotent response. Nogo trials, in which the participant was instructed not to respond, represented the other 25%. A depiction of this task is shown below in Fig. 1.

#### AX continuous performance task (proactive/reactive control)

The AX-CPT task was adapted from the task described by MacDonald and Carter^9^. Instead of being shown one letter, as in the Go/Nogo task, participants were shown pairs of letters in this task. The four types of pairs were “AX”, “AY”, “BX”, and “BY”, with the “AX” pair being the special pair with distinct instructions from the rest. The task began with a 500 ms fixation stimulus in the middle of the screen, as shown in Fig. 1. Then the cue was presented (first letter of the pair) for 400 ms and colored light blue to let participants know when a new pair began. After the first letter (cue) was shown, the participant was allowed an additional 1300 ms to respond with response button #1, after which a fixation stimulus was shown for 2000 ms. This was the case for every trial, regardless of the condition. After this fixation stimulus, the second letter within the pair was shown in white for 400 ms. This stimulus was termed the probe. After the probe was shown, the participant had an additional 1300 ms to respond with either response button #1 or #5 (explained below), after which another fixation stimulus was displayed for an inter-trial interval that varied from 1000 to 2000 ms. If the second letter was an “X” preceded by an “A” (making it an “AX” pair), then participants responded to the letter “X” with the button labeled #5. Hence, for “AX” trials, participants responded with button #1 after the “A” letter and with button #5 after the “X” letter, as shown in Fig. 1. These “AX” trials comprised 70% of the trials for each block of the AX-CPT, while the other three trial types comprised the remaining 30% equally (10% each condition). If the second letter was a “Y” preceded by an “A” (making it an “AY” pair), then participants responded to the “Y” letter with the button labeled #1. So, for “AY” pairs, the participant was expected to respond with button #1 after “A” and button #1 after “Y”. During these “AY” trials, participants were first primed with the “A” cue, and since the “AX” condition was prepotent, they expected that the second letter would be “X”. However, since the second letter was “Y” in “AY” trials, participants had to react to the “Y” probe and change their second response to button #1. This particular trial requires reactive control. During trials with a “B” cue, participants were supposed to respond with button #1 for both cue and probe segments. In these “B” trials, participants had to remember that they had been cued with the letter “B” when responding during the probe period. Hence, keeping in mind that they saw a “B” letter for the cue required proactive control.

#### Global/local Navon task (response Conflict)

The task eliciting response conflict was a modified Flanker task that presented Navon Letters^34^ as the conflict inducing stimulus rather than arrows, thereby increasing the difficulty of the task. In this task, participants were shown a big letter (Global) composed of smaller letters (Local). Sometimes the big and small letters matched (“congruent”) and sometimes the big and small letters did not match (“incongruent”; *See* Fig. 1). The conditions in this task were 1) big “H” made of small “S” (“incongruent”), 2) big “H” made of small “H” (“congruent”), 3) big “S” made of small “H” (“incongruent”), and 4) big "S" made of small "S" (congruent). During each trial, participants were first shown the local/global indicator for 2000 ms, which was the word “Big” or “Small”. This let participants know if they should respond to the larger, overall letter shape or to the smaller letter, respectively. Then participants were shown the actual letter stimulus for 200 ms. After this interval, participants were shown a fixation cue for 1100 ms, during which they responded to the previous stimulus. Regardless of the “Big” or “Small” conditions, if the participant believed the correct response was the letter “H”, they responded by pushing button #1. If the correct response was believed to be “S”, participants responded by pushing button #5. Next, another fixation stimulus was shown for an inter-trial interval that varied from 0 to 500 ms. For this task, the “incongruent” trials were of particular interest, since the participant was required to resolve the conflicting “S” and “H” stimuli in order to respond correctly. Hence, this task tested the participants’ ability to resolve response conflict.

### EEG preprocessing and analysis

All EEG recordings were completed using a 129-channel HydroCel Geodesic Sensor Net with a potassium chloride solution to facilitate the electrical readings. The recordings were sampled at 1000 Hz using EGI Netstation Acquisition software (Electrical Geodesics, Inc., Eugene, Oregon). Data acquisition began after impedances were below 50 kΩ. All channels were referenced to the Cz electrode during data acquisition but re-referenced to an average of all electrodes offline for data analysis. After EEG data acquisition was complete, data processing was implemented using EEGLAB^35^ running in MATLAB R2019b.

The EEG data were pre-processed as follows. Data were band-passed between 0.1 Hz and 35 Hz using a zero-phase Hamming windowed-sinc FIR filter, then downsampled to 125 Hz. Noisy channels were rejected if the joint probability of that channel’s data and all channel data exceeded four standard deviations. Data were then epoched to form stimulus-locked segments ranging from 2500 ms pre-stimulus to 3000 ms post-stimulus. This large range was chosen to account for edge artifacts created during wavelet convolution^36^. The time-locked stimuli for the separate cognitive control strategies were: the “A” and “B” cues for proactive control, the “X” probe for “AX” trials and the “Y” probe for “AY” trials for reactive control, the “X” stimulus (Nogo) and all other letters (Go) for inhibitory control, and the congruent and incongruent stimuli for resolving response conflict. Thus, for each of four cognitive control strategies, we separately epoched and examined trials that required low amounts of control (control trials) and trials that required high amounts of control (target trials). Epochs were then mean centered in preparation for artifact detection. Independent component analysis (ICA) was performed on the data using the *runica()* EEGLAB function^37^ and artifactual ICs were tagged using SASICA^38^, which detected artifact ICs using autocorrelation statistics, the ADJUST plugin^39^, and correlation with EOG channels. ICs were additionally labeled using the ICLabel plugin^40^, and components labeled as artifactual by SASICA or classified as artifactual by ICLabel were removed from the data. Any residual ocular artifacts were removed by thresholding frontal channels at ± 140 μV, and remaining artifacts exceeding ± 140 μV were removed via trial-by-trial channel interpolation^41^. Channels that were artifactual on more than 30% of trials were deleted outright, and any epoch with more than 10% of remaining channels deemed bad was rejected outright. Removed channels were interpolated using spherical interpolation and data were average referenced. If any subject completed less than 10 correct, clean trials for any cognitive control condition, they were removed from further analysis. The 176-subject sample only includes the subjects that met these criteria. See Table 1 for statistics describing trial counts in each condition.

**Table 1:** Trial counts for each condition in the current study.

Strategy	Mean	SD	Min	Max
Proactive-control	285.8864	33.0369	168	334
Proactive-target	68.2784	8.8393	31	79
Reactive-control	266.8864	28.1471	160	301
Reactive-target	20.2898	6.7672	10	37
Inhibitory-control	164.6023	14.6981	91	180
Inhibitory-target	33.2216	8.2595	15	55
Conflict-control	114.1705	19.0687	43	144
Conflict-target	100.6818	18.6768	39	142

**Figure 2:**
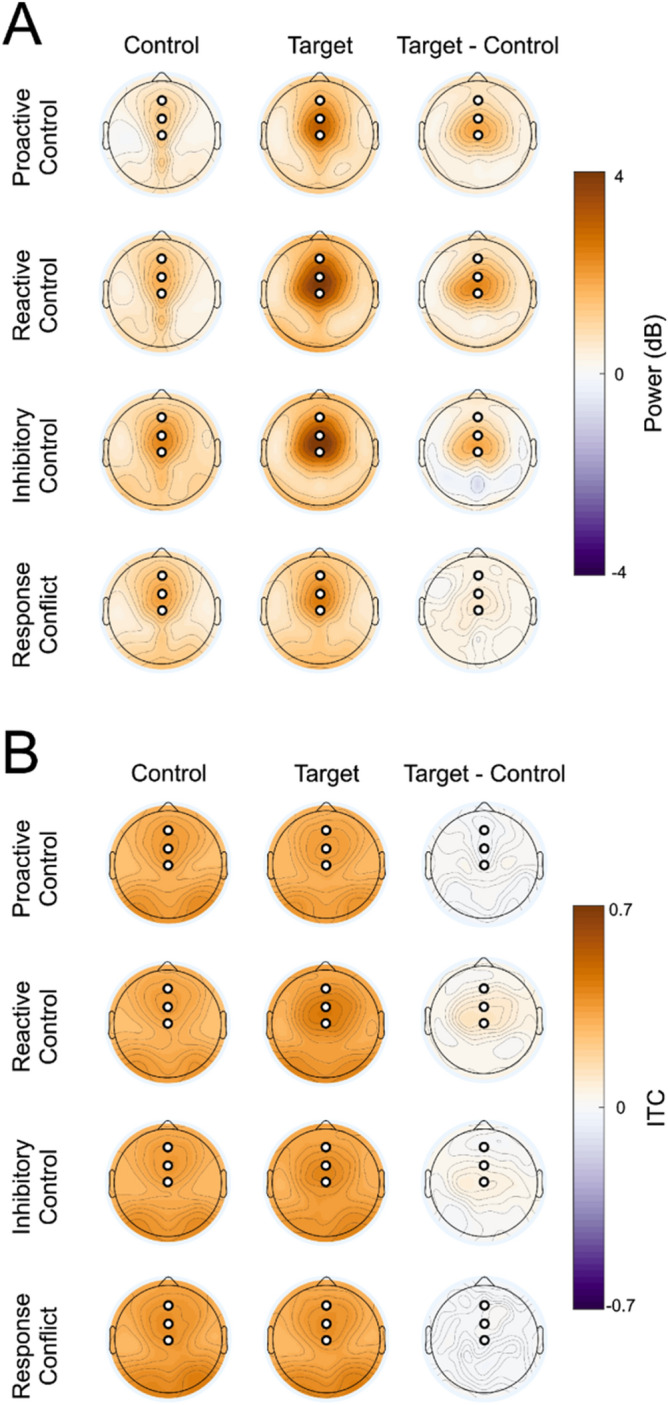
Topographic plots for each cognitive control strategy generated by convolving the EEG data with Morlet wavelets at each sensor and displaying the average (**A**) ERSP and (**B**) ITC value for that electrode within the time range of 200–450 ms post-stimulus. White circles indicate the sensors that were selected for analysis (top to bottom: Fz, FCz, Cz). Low control indicates control trials and high control indicates target trials.

Following pre-processing, the EEG data was ready for time–frequency analysis. Using EEGLAB’s *newtimef()* function, individual correct trials were convolved with a series of complex Morlet wavelets, focusing on 25 linearly-spaced frequencies ranging from 1 to 25 Hz, to create a time–frequency depiction of the EEG signal. A complex Morlet wavelet is a complex, Gaussian-tapered, sine wave represented by the equation ${e}^{2\pi tf}{e}^{{t}^{2}/(2{\sigma }^{2})}$, where t represents time, f represents frequency, and σ represents the width of each frequency band according to s/(2πf). In this sub-formula, s represents the number of cycles. An adaptive number of cycles was used in this analysis, in which 3 cycles were used at 1 Hz and the number of cycles was increased equally until 10 cycles were used at 25 Hz. This improves the temporal resolution of the analysis at low frequencies and the frequency resolution of the analysis at high frequencies^36^. The baseline measurement, used in the calculation of spectral power in decibels (dB), was taken from − 500 to − 200 ms pre-stimulus. Decibel power was calculated via the formula 10 × log10[power(t)/power(baseline)], which used the power that was calculated previously by the formula real[z(t)]^2^  + imag[z(t)]^2^. Z(t) represented the magnitude of the analytical, convolved signal. ITC was calculated using the formula $\left|\frac{1}{n}{\sum }_{x=1}^{n}{e}^{i{\phi }_{x}}\right|$, where n is the number of trials for each time and frequency band and ${\phi }_{x}$ is the phase angle at the particular time–frequency point. The phase angle is the angle formed from the real z(t) signal and the imaginary z(t) signal with respect to the real axis, calculated via the function arctan[imag[z(t)]/real[z(t)]]. Since ITC analyses can be confounded by condition differences in the number of trials that comprise the data, we used a subsampling procedure where we first detected the condition (out of 8; control and target trials for each of four strategies) with the lowest trial counts within each subject, then randomly subsampled that number of trials from each condition. Because this study focused on cognitive control-related theta, which is maximal over frontal midline sensors, the electrodes that were chosen for time–frequency analysis were sensors 6 (FCz), 11 (Fz), and 129 (Cz). These electrodes and time periods (200–450 ms) were selected a priori based on reports that theta power is maximal over frontal midline sensors, and were verified based on examination of theta power topographic plots (averaged over all conditions). Topographic maps for each experimental condition (Fig. 2) show a clear frontal midline focus of theta power and ITC, as expected based on previous literature.

### Statistical analysis

For each cognitive control strategy, we use the notation "control" trials to reference the low-control comparison trial type (Go/Nogo task: go trials; AX-CPT: AX trials; Global/Local task: congruent trials), and we use the notation "target" trials to reference the high-control trial type of interest (Go/Nogo task: nogo trials; AX-CPT: AY trials [reactive control, probe-locked]; B trials [proactive control, cue-locked]; Global/Local task: incongruent trials).

For statistical analysis of behavioral data, reaction times and accuracies were averaged over all single trials within each condition. Since two of the cognitive control strategies (AY—reactive control, BX—proactive control) had the same control comparison condition (AX), we could not enter all comparisons in a single ANOVA. Instead, we used paired *t*-tests to compare accuracy within control and target conditions for each strategy, then calculated the interference effect for each cognitive control strategy (control accuracy—target accuracy) and analyzed these interference scores using a repeated-measures ANOVA with a single factor (cognitive control strategy). We used the same approach to analyze reaction times, except that analysis of reaction times did not include the inhibitory control strategy because subjects did not respond on nogo trials.

For statistical analysis of EEG data, frontal midline theta activation and ITC for each condition were computed as the average activation (dB spectral power) or ITC per subject within 4–8 Hz frequency and 200–450 ms time post-stimulus. EEG data were exported and statistically analyzed in SPSS (https://www.ibm.com/products/spss-statistics). Frontal midline theta ERSP and ITC were compared across the four cognitive control strategies using two 4(Cognitive Control Strategy: reactive control, proactive control, inhibitory control, response conflict)-by-2(Trial Type: target, control) repeated-measures ANOVAs on theta power and theta ITC.

For all analyses, we applied a Bonferroni correction to all post-hoc analyses. Because some analyses violated the Sphericity assumption, all omnibus ANOVA results reported here had the Greenhouse–Geisser correction applied. Post hoc analysis of significant ANOVA effects used the SPSS EMMEANS command.

We additionally completed an individual differences analysis by examining whether greater cognitive control-related theta power or phase-locking was related to inter-individual differences in RT or accuracy across the four strategies. For this analysis, we first residualized accuracy, RT, theta power, and theta ITC for all target trials by their respective control trials, thus isolating cognitive control-related effects without the negative psychometric properties associated with difference scores for individual differences analysis with EEG data^42^. For each of four strategies, we then correlated the residualized accuracy rates with the residualized theta power and ITC metrics, and for each of three strategies (excluding inhibitory control), we correlated the residualized RTs with the residualized theta power and ITC. All correlation analyses used Pearson correlations.

### EEG decoding analysis

The previous analyses allow us to isolate condition differences in frontal midline theta activation, but tests for differences do not provide strong evidence for generalizability of neural representations across multiple cognitive control strategies. Thus, we employed a strategy of decoding control from target trials for each of four cognitive control strategies, using multivariate pattern classifiers trained on each of the four strategies. Similar decoding approaches have shown considerable utility in prior EEG analyses^43,44^. Since classifiers were trained and tested on each combination of two cognitive control strategies, we used a total of 16 decoding analyses (since we also ran analyses decoding control from target trials using a classifier trained on separate trials from the same strategy). The decoding analyses used the average theta power time courses from each of the three frontal midline sensors used in the prior analyses. To ensure that classification accuracy was unbiased, we equalized trial counts between control and target trials for each training and testing set using a random subsampling approach (this also resulted in a chance decoding accuracy of exactly 0.5). We trained and tested each classifier using linear discriminant analysis on single trials of data for each subject. We evaluated decoding quality using fivefold cross-validation, thus guaranteeing that train and test data had no overlap, even when training and testing classifiers on the same strategy. This analysis was run at each time point from 0 to 1000 ms in 20 ms steps. Within each subject, this entire analysis was run three times and the decoding accuracy averaged, thus reducing the influence of random variations dependent on random subsampling and the assignment of trials to cross-validation folds. For each of 16 decoding analyses and each time point, single-subject average decoding accuracies (averaged over 3 iterations of 5 cross-validation folds) were tested against a null hypothesis mean of 0.5 (chance accuracy) using one-sample *t*-tests, with control for multiple comparisons established via control of the false discovery rate^45^ (applied to concatenated *p*-values from all 16 decoding analyses, to provide stringent control of the false discovery rate).

## Results

### Behavioral

For accuracy rates, paired *t*-tests revealed a significant accuracy decrease for target conditions compared to control conditions for all four cognitive control strategies, proactive control mean interference = 4.24%, *t*(175) = 6.32, *p* < 0.001, reactive control mean interference = 40.49%, *t*(175) = 34.71, *p* < 0.001, inhibitory control mean interference = 38.9%, *t*(175) = 41.11, *p* < 0.001, response conflict mean interference = 11.3%, *t*(175) = 18.53, *p* < 0.001. A repeated-measures ANOVA on accuracy interference scores with one factor (cognitive control strategy) showed a significant effect of cognitive control strategy, *F*(3,525) = 536.50, *p* < 0.001, η^2^ = 0.75. Post hoc testing showed that inhibitory control and reactive control both had higher interference scores than proactive control and response conflict, while response conflict had higher interference than proactive control (all *p* < 0.001).

**Figure 3:**
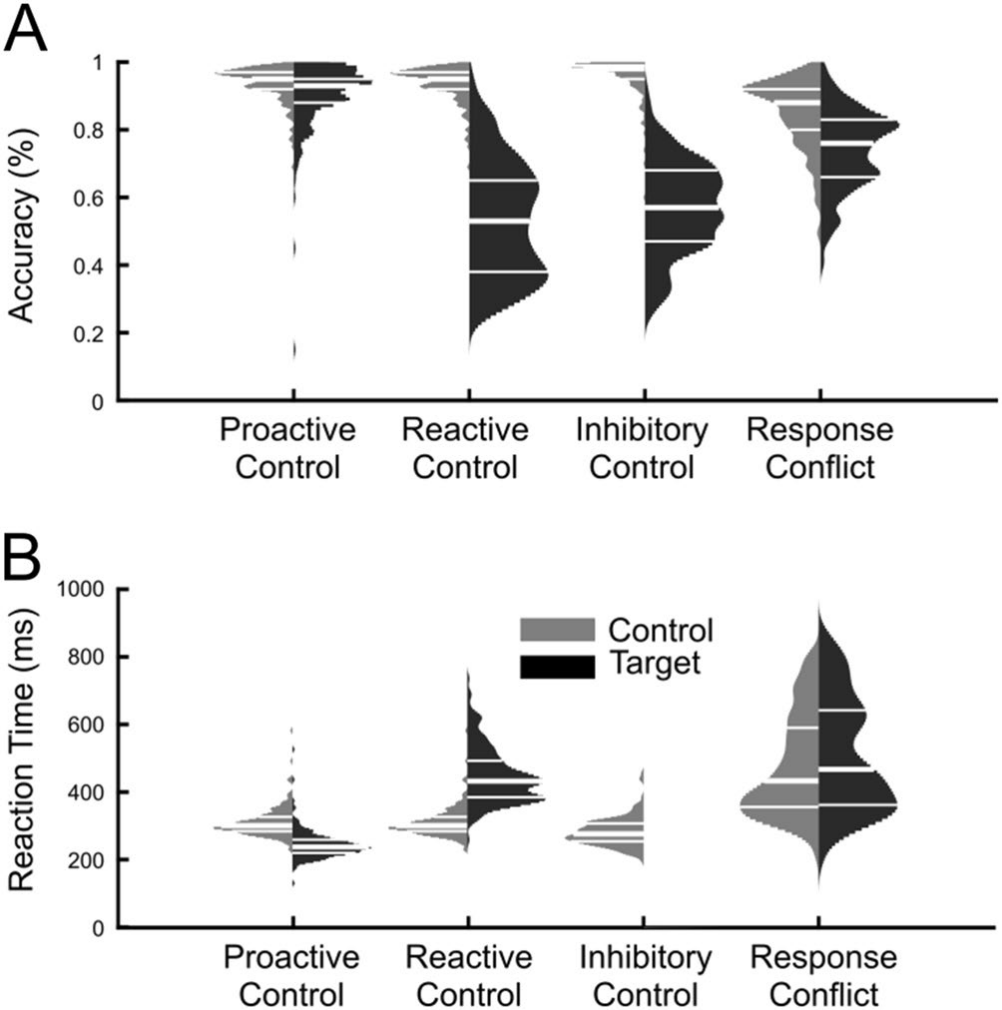
Distribution plots (violin plots) of accuracy and reaction times across four different cognitive control strategies (proactive control, reactive control, inhibitory control, and response conflict). Note that in the RT plot, target inhibitory control trials do not have any RT because the correct response in this strategy was to inhibit a response. White horizontal lines mark 25th, 50th, and 75th percentiles of data distribution.

For reaction times, paired *t*-tests revealed a significant RT increase for target conditions compared to control conditions for two of three cognitive control strategies, reactive control mean interference = 138.99 ms, *t*(175) = 28.49, *p* < 0.001, response conflict mean interference = 32.11 ms, *t*(175) = 11.52, *p* < 0.001. Proactive control showed an opposite effect, such that RTs were significantly faster for high control (BX) trials compared to low control (AX) trials, mean difference = 65.55 ms, *t*(175) = 27.21, *p* < 0.001. A repeated-measures ANOVA on RT interference scores with one factor (cognitive control strategy) showed a significant effect of cognitive control strategy, *F*(2,350) = 922.33, *p* < 0.001, η^2^ = 0.84. Post hoc testing showed that all three strategies differed from each other in interference; reactive control had higher interference than proactive control and response conflict, while response conflict had higher interference than proactive control (all *p* < 0.001). See Fig. 3 for a summary of behavioral results.

### Frontal midline theta power and ITC ANALYSES

**Figure 4:**
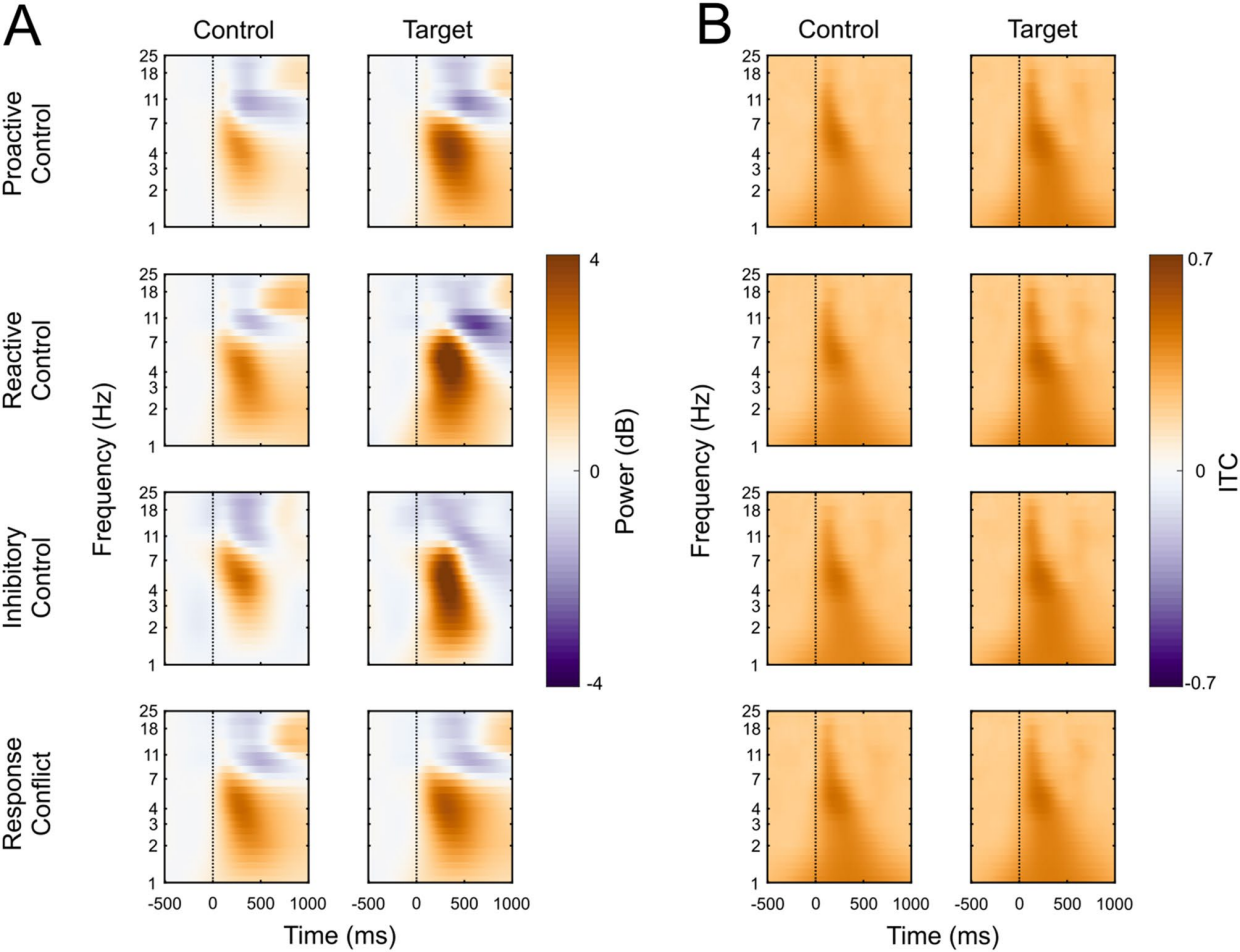
(**A**) Event-related spectral perturbation (ERSP) and (**B**) Inter-Trial Coherence (ITC) plots for each trial type and task epoched from − 500 ms to 1000 ms in relation to the time-locking stimulus. The baseline was taken from − 500 to − 200 ms to compute the event-related power in decibels. Data are plotted on a logarithmic frequency axis to better visualize low-frequency activity.

**Figure 5:**
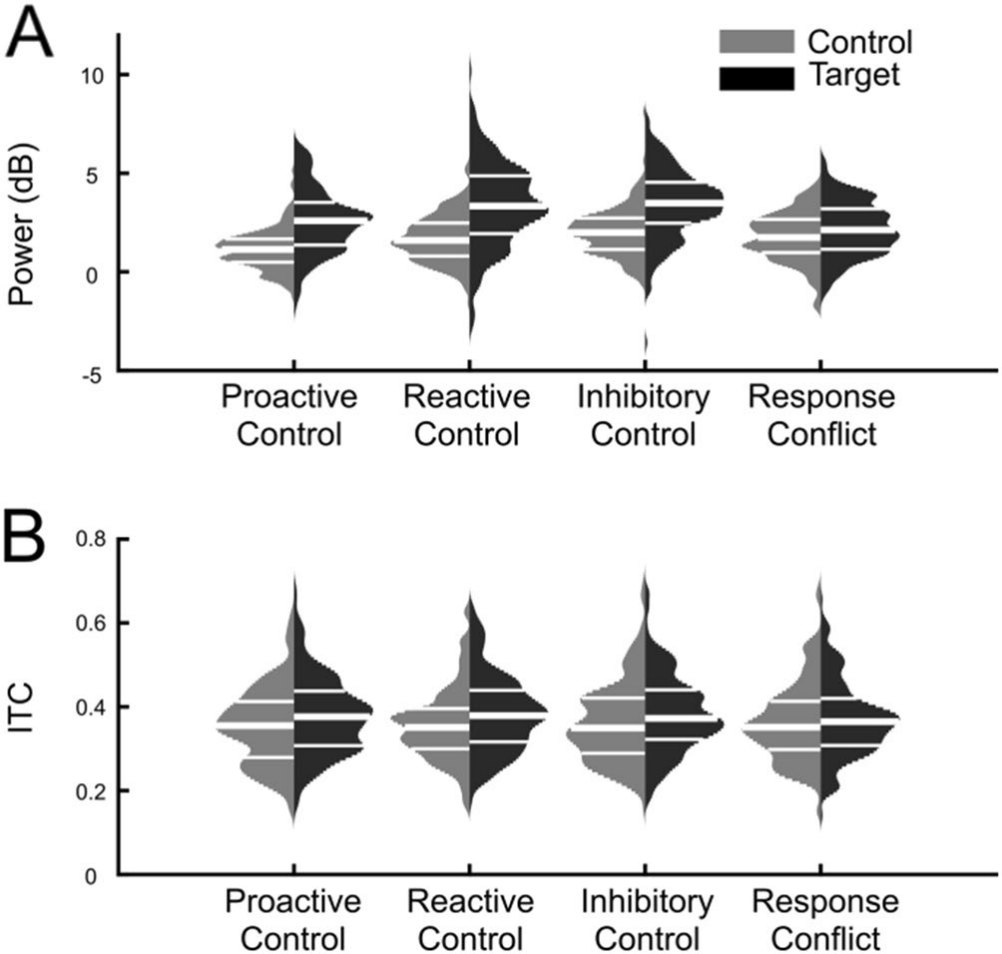
Distribution plots (violin plots) of condition means for theta power and ITC across the four cognitive control strategies. White horizontal lines mark 25th, 50th, and 75th percentiles of data distribution.

Analyses of theta power revealed a main effect of Cognitive Control Strategy, *F*(3,525) = 69.78, *p* < 0.001, η^2^ = 0.29, and a main effect of Trial Type, *F*(1,175) = 453.61, *p* < 0.001, η^2^ = 0.72, which were both subsumed by a Cognitive-Control Strategy-by-Trial Type interaction, *F*(3,525) = 48.04, *p* < 0.001, η^2^ = 0.22. Bonferroni-corrected contrasts revealed that all target trial types were significantly higher in theta power than control trial types (*p* < 0.001). For control trials, contrasts revealed that go trials showed more theta power than the control trials for reactive control, proactive control, and response conflict (*p* < 0.001). Additionally, for control trials, response conflict (*p* < 0.001) and reactive control (*p* < 0.001) showed greater power than proactive control. For target trials, reactive control and inhibitory control showed more power than proactive control and response conflict (all *p* < 0.001), and proactive control showed more power than response conflict (*p* < 0.001). For frontal midline theta ITC, the ANOVA indicated only a main effect of Trial Type, *F*(1,175) = 35.15, *p* < 0.001, η^2^ = 0.17, such that target trials had higher theta ITC than control trials (*p* < 0.001). Overall, the pattern of results indicates that all target conditions resulted in increased theta power and ITC compared to control trials, and theta power was overall highest for inhibitory control and reactive control strategies. A graphical representation of these comparisons is shown in Figs. 4 and 5.

### Individual difference analysis

We used Pearson correlations to examine whether frontal midline theta was related to individual differences in task performance (accuracy & RT). These analyses returned few concordant results; we found only that for reactive control both theta ITC correlated with accuracy rates (*p* = 0.003) and theta power correlated with RTs (*p* = 0.02). For response conflict, theta ITC correlated with RTs (*p* = 0.01). Full results of correlation analyses are shown in Table 2.

**Table 2:** Results of individual differences analysis (correlating theta power and ITC with accuracy and RT for each cognitive control strategy).

Strategy	ACC	RT
Proactive theta power (dB)	*r* = .14, *p* = .14	*r* = .07, *p* = .33
Reactive theta power (dB)	*r* = −.02, *p* = .79	*r* = .18, *p* = .02*
Inhibitory theta power (dB)	*r* = .12, *p* = .11	#
Response Conflict theta power (dB)	*r* = −.03, *p* = .72	*r* = .016, *p* = .83
Proactive theta ITC	*r* = .1, *p* = .19	*r* = .06, *p* = .46
Reactive theta ITC	*r* = −.22, *p* = .003**	*r* = .04, *p* = .57
Inhibitory theta ITC	*r* = .11, *p* = .17	#
Response Conflict theta ITC	*r* = .05, *p* = .50	*r* = .19, *p* = .01*

*p < .05, **p < .01.

Correlations represent Pearson correlations with 174 degrees of freedom.

### Theta decoding results

**Figure 6:**
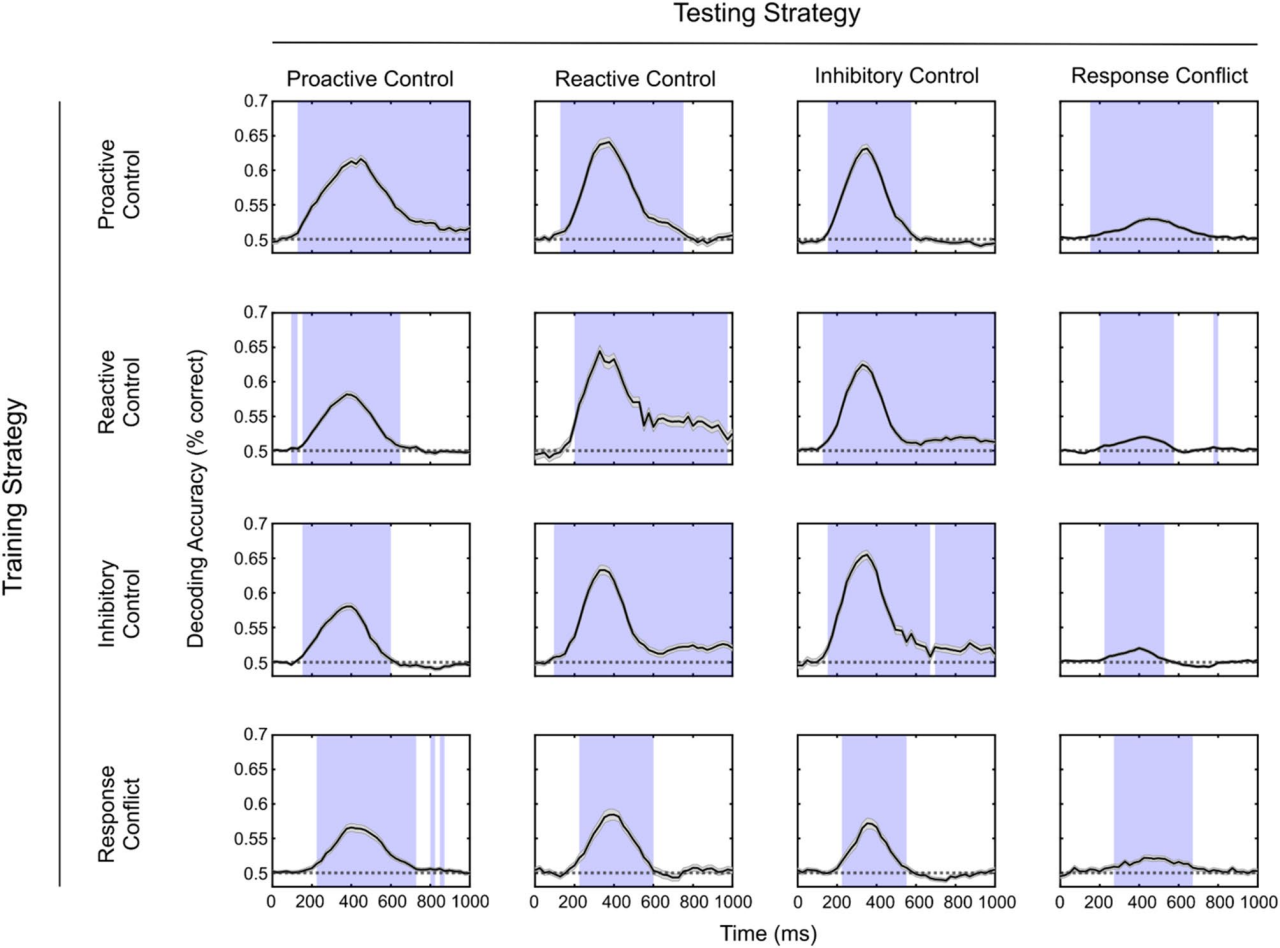
Average accuracy of decoding for each combination of train and test strategies. Transparent shade around the black lines indicates standard error, and the transparent background shade encloses samples where decoding accuracy was significantly different from chance (0.5). Results indicated that control and target trials could reliably be decoded, even when classifiers were trained exclusively to distinguish control from target trials for a different cognitive control strategy.

We examined whether frontal midline theta activation generalizes representations of the need for cognitive control between multiple cognitive control strategies using decoding analyses. Specifically, we evaluated whether we could decode control from target trials for each strategy, using pattern classifiers trained on each of the cognitive control strategies. Within each cognitive control strategy, we trained and tested pattern classifiers on separate sets of trials using fivefold cross-validation to prevent any bias in decoding evaluation, and for each strategy, we were able to successfully decode control from target trials with greater-than-chance accuracy. In an analysis that directly informs the question of generalizability of theta representations of the need for cognitive control, we used a similar strategy to decode control from target trials in each strategy, using only pattern classifiers trained to discriminate a different cognitive control strategy. We found that, for each combination of strategies, decoding accuracy was significantly greater than chance during time periods between 200 and 450 ms post-stimulus. Concatenated sample-by-sample *p-*values resulting from testing decoding accuracy against a null hypothesis of 0.5 (chance decoding) were corrected by control of the false discovery rate, thus conclusively demonstrating that frontal midline theta generalizes the need for cognitive control across multiple cognitive control strategies. Results of decoding analysis are shown in Fig. 6.

## Discussion

In the current study, we compared theta power and inter-trial coherence (during the time frame of the N2) across four different cognitive control strategies: proactive control, reactive control, inhibitory control, and resolving response conflict. Results revealed relatively high levels of frontal midline theta-band (4–8 Hz) power and inter-trial coherence within the timeframe of 200–450 ms post-stimulus across all cognitive control strategies, suggesting an underlying mechanism for executing all four of the cognitive control strategies. These results are in alignment with many previous studies on cognitive control and its relation with theta-band activity^21,26,28,46^. This result supports the hypothesis that theta power (and phase-locking) increases are a common substrate, or a lingua franca, for cognitive control^13^.

As predicted, we found significantly higher theta-band power for target trials than control trials for all four cognitive control strategies. Similarly, we found significantly higher target trial ITC values than for control trials for each of the four cognitive control strategies. Furthermore, the fact that we found worse performance accuracy (for all four strategies) and longer reaction times (except for proactive control) for target trials than control trials, suggests that higher neural activation is associated with processing the need for cognitive control. Additionally, these results indicate the experimental procedure was designed and implemented in agreement with previous cognitive control studies.

Interestingly, theta-band ERSP values were highest in the reactive control and inhibitory control target trial types. This additional neural demand coincides with a larger drop in accuracy rates (for reactive control and inhibitory control) and a larger increase in reaction time (for reactive control) between the control and target trials, compared with the other strategies. Combined, these results suggest that reactive control and inhibitory control mechanisms exert a larger demand for frontal midline theta-band activation (as seen through the ERSP analyses), compared to other cognitive control mechanisms. These findings are interesting because both reactive control and inhibitory control involve overriding an erroneous dominant response, a specific type of control known to require activation of the anterior cingulate cortex (ACC) and lateral prefrontal cortex^8,47^. Given the commonalities between inhibitory and reactive control, it is possible that these cognitive control strategies have common underlying physiological mechanisms. This seems particularly likely since our generalizability analysis using decoding demonstrated that classifiers trained to discriminate reactive control could just as easily discriminate inhibitory control, and vice-versa. Based on our results, we suggest that cognitive control strategies that override a prepotent response depend on theta activity time-locked to the stimuli that initiates overriding the dominant response.

The consistency in results across statistical analyses, for example with reactive control and inhibitory control having the largest theta power, accuracy interference score, and reaction time interference score, could signify the beginning of a numerical framework for comparing cognitive control strategies on their theta power and ITC values. It has been suggested that theta power values might be a more sensitive index of between-condition differences than ERP analyses^13^, so future work in cognitive neuroscience could focus on how the brain specifically uses theta power to interpret and coordinate information. It has been theorized that transient theta dynamics are the basis for coordinating distant neural populations for flexible communication and execution of cognitive control^26^, with frontal midline brain regions functioning as nodes for monitoring conflict and directing other brain regions for goal-oriented behavioral adaptations to conflict. For instance, the midcingulate cortex (MCC) is heavily connected with cortical and subcortical brain regions, and it has been theorized that the MCC acts as a hub for organizing brain systems across large spatial distances through frontal midline theta oscillations^2^. Understanding the nature of these theta dynamics in terms of their power and phase is important, as it likely impacts forthcoming neural communications and computations that initiate the action selection and action production processes^2^. In fact, it has already been shown that increased theta power is associated with enhanced coupling between single neuron spikes in rats^48^ and monkeys^49^. Hence, as stated previously, this study provides support for the theory that frontal midline theta oscillatory activity is key to organizing neural processes underlying several strategies of cognitive control. However, what remains to be understood is precisely how these strategies are differentiated by the brain to result in rapid integration of information and communication with distal networks for goal-driven decision making. It has been suggested that synchronized changes in the phase angle of neural oscillatory activity can create time frames for segregating cortical populations^2^, one explanation of how varying the phase angle of neural communication allows the brain to execute distinct tasks. This still needs to be verified further; thus, this study prompts further investigation into the distinct mechanisms that underlie the various cognitive control strategies studied in cognitive neuroscience.

Standard ANOVA-based analyses of theta power demonstrated notable condition differences between the various cognitive control strategies, while also demonstrating increases in theta power for all target trial types (compared to control trials). Thus, our results suggest that theta power simultaneously generalizes the need for cognitive control, while also differentiating specific cognitive control strategies. However, statistical tests for differences by design can only quantify whether or not two measures are different, and thus have limited utility in testing whether theta-band representations of cognitive control truly generalize across multiple cognitive control strategies. Therefore, in a series of followup analyses we turned to decoding analysis, which has previously shown high utility for use with EEG data^43,44^. We demonstrate in this analysis that we can successfully distinguish control from target trials for each of the four cognitive control strategies using only theta-band activation at three frontal midline sensors. Much more importantly, we showed that this decoding success persisted when we trained the classifier on one strategy and tested it on a different strategy, for every combination of strategies. As an example, this demonstrates that go trials could be distinguished from no-go trials (inhibitory control) by a classifier that was only trained to distinguish congruent from incongruent trials (response conflict). This provides the first true evidence that frontal midline theta power generalizes representations of the need for cognitive control across multiple cognitive strategies, since prior analyses of theta across multiple cognitive control strategies have always relied on tests for condition differences. Thus, we conclusively establish that, while theta power differentiates between cognitive control strategies, theta power simultaneously generalizes the need for cognitive control across multiple cognitive control strategies.

Given the large sample size in the current study, we were well-powered to examine whether frontal midline theta (power and ITC) correlated with individual differences in cognitive performance. The results of this individual differences comparison revealed a sparse pattern of results. The only significant theta power correlation was a relationship between increased theta power and slower reaction times for reactive control trials. This result suggests that individuals who recruit greater cognitive control-related theta power in reactive control conditions respond in a slower, more careful fashion, and as such, theta power might represent a mechanism that actively slows responding during the need for cognitive control. A similar result has been demonstrated using within-subject single-trial analysis as well^28^. Individual differences results additionally demonstrated that increased theta phase-locking correlated with decreases in accuracy for reactive control trials, and with slower reaction times for response conflict. These effects generally suggest that increases in theta phase-locking correlate with decreased cognitive control ability across individuals, at least for cognitive control strategies that require last-minute selection of a competing response (reactive control and response conflict).

This study verified that many cognitive control mechanisms depend on frontal midline theta-band neural power and phase-locking to external stimuli, while noting condition differences in theta power between several cognitive control strategies. This raises the idea that at least part of the differentiating mechanism the brain employs to execute appropriate cognitive control mechanisms involves theta-band power. The results of this study emphasize that increased power in the theta frequency band is especially important for cognitive control strategies that require overriding a prepotent response (reactive control and inhibitory control), while increased inter-trial theta phase-locking appears equally important for all cognitive control strategies. It is important to note that our experimental design only addresses separate cognitive control strategies in comparatively low-control and high-control scenarios. Our data do not address cognitive effort, a construct often conflated with cognitive control that refers to the degree of engagement with which one performs a cognitively demanding task^50^. In order to dissociate cognitive control from cognitive effort, the tasks would need to incorporate a reinforcer that could be used by participants to evaluate the reward from investing cognitive effort into the task at hand, according to the expected value of control theory^51,52^. The inability to dissociate cognitive effort from cognitive control is a limitation of this study. Notably, a recent study from our group demonstrated that reinforcement modulates frontal midline brain activity and behavior in a cognitive control task, but this study only included a single cognitive control strategy^53^. Future directions should incorporate a reinforcement element with the multiple cognitive control strategies compared in the current study. This would contribute to the active area of research that aims to elucidate the dorsal anterior cingulate cortex’s function in regulating cognitive control, particularly with respect to how this region might incorporate reward into the evaluation of cognitive control^51,52^.

Future work also should incorporate more accurate spatial information from fMRI, ECoG, deep brain stimulation, and cellular neuroscience studies in order to gain a fuller understanding of functional connectivity, specifically how frontal midline theta power and phase dynamics are used to communicate between, coordinate, and segregate neuronal populations. The cortical generators of theta rhythms are an active area of current research, and current literature supports a multitude of potential theta generators^1,14,15,54,55^. Investigating the sources of frontal theta might be particularly informative, as an emerging literature suggests theta rhythms might organize distributed patterns of prefrontal connectivity (i.e. between multiple different prefrontal generators)^2^. For instance, 6 Hz (i.e., theta band) transcranial alternating current stimulation (tACS) applied to the dorsolateral prefrontal cortex (DLPFC) was shown to reduce nodal efficiency with the dorsal ACC (dACC) during resting state fMRI^56^. In a separate study, transcranial direct current stimulation (tDCS) of the DLPFC reduced resting EEG theta power (relative to baseline) in frontal-midline regions^57^. While the current study did not include a causal manipulation (i.e. using TMS or tD/ACS) or source localization of electrocortical activity, this study was notably the first to show that frontal midline theta representations of the need for cognitive control generalize between multiple cognitive control strategies including proactive (planned) control (using decoding analyses), thus adding to our understanding of scalp-level theta as a generalizable marker of the need for cognitive control. Future work should examine whether this generalizability extends to source-level theta activation within the frontoparietal network.

## Conclusion

This study begins to differentiate cognitive control mechanisms on their respective amount of frontal midline theta power at 200–450 ms post-stimulus, or around the N2 interval. We established that cognitive control strategies that require overriding a dominant (prepotent) response (namely, reactive control and inhibitory control) reveal the highest theta power with greatest reductions in behavioral performance, suggesting that theta-band activity might be a driving force underlying these cognitive control strategies. By providing a differential analysis of the various cognitive control strategies, we gained more insight into the neurophysiological basis of cognitive control and how this basis varies amongst strategies. Hence, this research adds value to forthcoming functional connectivity studies as they try to further understand how neural populations operate to achieve cognitive control. Understanding the underlying mechanisms of cognitive control in a controlled setting could provide more information about the underlying mechanisms of clinical disorders, such as attention deficit hyperactivity disorder, obsessive compulsive disorder^58^, anxiety disorders^1^, and schizophrenia^9,59^, and for developing novel treatments for these conditions.
